# Gigantic Vortical Dichroism and Handedness-Dependent Optical Response in Spiral Metamaterials

**DOI:** 10.3390/nano16010065

**Published:** 2026-01-01

**Authors:** Kangzhun Peng, Hengyue Luo, Shiqi Luo, Zhi-Yuan Li, Wenyao Liang

**Affiliations:** School of Physics and Optoelectronics, South China University of Technology, Guangzhou 510640, China

**Keywords:** chiral optical response, spiral metamaterial, vortical dichroism, orbital angular momentum

## Abstract

Light carrying orbital angular momentum (OAM) has emerged as a promising tool for manipulating light–matter interactions, providing an additional degree of freedom to explore chiral-optical phenomena at the nanoscale. When such vortex beams interact with chiral metamaterials, a unique phenomenon of optical asymmetry known as vortical dichroism (VD) arises. Nevertheless, most existing chiral metamaterials exhibit limited VD responses, and the underlying physical mechanisms are yet to be fully clarified. In this work, we propose three-dimensional spiral metamaterials that achieve gigantic VD effect. This pronounced VD effect originates from the intrinsic coupling between the spiral structure and the chirality inherent to optical vortices, which leads to strongly asymmetric scattering intensities for left- and right-handed OAM beams of opposite topological charges. Numerical simulations confirm a remarkable VD value of 0.69. Further analysis of electric field distributions reveals that the asymmetric VD response stems from a handedness-dependent excitation of distinct electromagnetic modes. For opposite handedness, spatial mode mismatch results in enhanced scattering. In contrast, matching handedness enables efficient energy coupling into a guided spiral mode, which suppresses scattering. These findings not only deepen the physical understanding of VD mechanisms but also establish a versatile platform for developing advanced chiral photonic devices and enhancing OAM-based light–matter interactions.

## 1. Introduction

Beyond carrying energy and linear momentum, light also possesses angular momentum, comprising spin angular momentum (SAM) and orbital angular momentum (OAM) [1]. SAM originates from polarization and takes two discrete states, including right- and left-handed circular polarization [2]. In contrast, the OAM arises from a helical phase in the light field. Vortex beams carrying OAM have spiral wavefronts with a phase factor of *e^ilϕ^* [3], where |*l*| represents the topological charge, determining the handedness and degree of spirality of the wavefront [4]. Unlike SAM, which is limited to two polarization states, OAM offers an unbounded set of degrees of freedom, opening up new possibilities for optical manipulation, information transmission, and imaging [5,6,7].

Chirality refers to the geometric property of a structure that it cannot be superimposed onto its mirror image by any combination of rotation or translation [8]. This property is commonly found in molecular systems, biomacromolecules, and artificially designed metamaterials. The optical response of chiral metamaterials is closely related to the angular momentum of incident light. Traditional chiral optical effects, such as circular dichroism (CD) [9], primarily result from the coupling between the SAM of light and the chiral medium. This manifests as differences in absorption, reflection, or transmission between left- and right-handed circularly polarized light [10,11]. However, recent studies have revealed that the OAM beam can also induce novel optical responses when interacting with chiral media [12]. When a vortex beam impinges on a chiral medium, its azimuth can couple to the chirality of the structure, thereby producing dissimilar scattering intensity for opposite topological charges. This phenomenon, known as vortical dichroism (VD), extends the chiral optical responses from the polarization dimension to the phase dimension, offering new degrees of freedom for research in chiral optics [13,14,15].

Despite the conceptual and experimental progress, most reported OAM-induced chiroptical effects remain weak. Interactions between vortex beams and natural chiral molecules typically produce negligible dichroic signals, owing to the size mismatch between the molecular scale and the optical vortex field. Even for artificial chiral metamaterials and plasmonic nanostructures, the VD effect is often limited to weak intensity [16,17,18]. As a result, gigantic chiral VD responses have been difficult to achieve. Overcoming these limitations requires carefully engineered chiral metamaterials that enhance light–matter interactions and to ensure effective spatial coupling with the optical vortex [19], thus enabling robust detection and modulation of OAM-driven chiral phenomena.

In this study, we propose three-dimensional spiral metamaterials to enhance the interaction between vortex beams and chiral metamaterials. The spiral metamaterials naturally possess intrinsic handedness, providing strong coupling with OAM-carrying light. By systematically optimizing the geometric parameters, the designed metamaterials support pronounced VD effect as high as 0.69. Analysis of the electric-field distributions reveals that the scattering asymmetry originates from handedness-dependent modal coupling mechanism. When the structural handedness opposes that of the vortex beam the spatial mode mismatch results in strong re-radiation and enhanced backscattering. Conversely the beam efficiently couples into a guided spiral mode when the handedness matches which diverts energy forward and effectively suppresses backscattering. The proposed approach offers a simple yet efficient way to amplify OAM-induced chiral optical responses, paving the way for novel chiral photonic devices, reconfigurable optical components, and advanced applications in optical communication and sensing.

## 2. Structure Design

**Figure 1:**
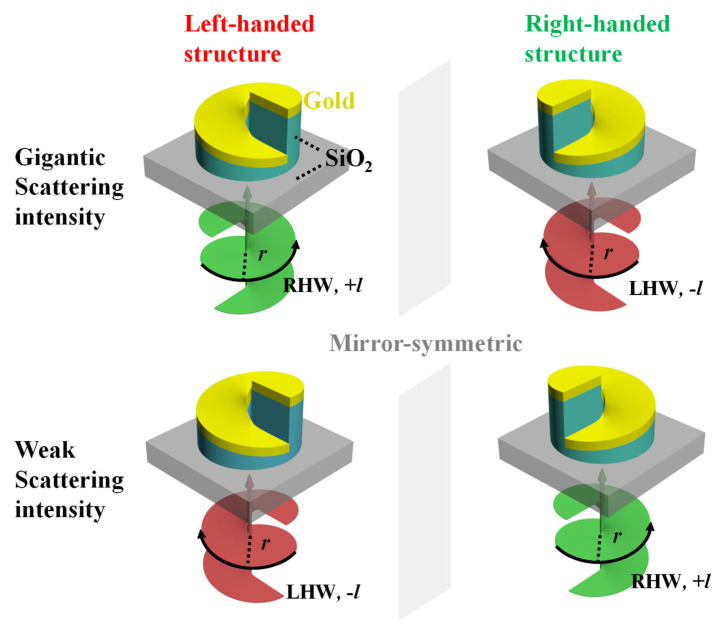
Mirror-symmetric left-handed and right-handed spiral metamaterials illuminated by vortex beams with opposite helical wavefronts (RHW, +*l*; LHW, −*l*). The asymmetric coupling between optical and structural handedness gives rise to distinct VD responses.

Figure 1 illustrates the VD effect observed when chiral spiral metamaterials are illuminated by vortex beams carrying opposite orbital angular momentum (OAM) states. The incident beams are denoted as left-handed helical wavefronts (LHW, −*l*) and right-handed helical wavefronts (RHW, +*l*). Each vortex beam exhibits a ring-shaped intensity distribution whose radial position depends on the topological charge *l*. The proposed structure consists of a top metallic spiral and a bottom dielectric spiral supported by a silicon dioxide (SiO_2_) substrate. Specifically, the top spiral structure is composed of gold (Au), while the bottom spiral part and the substrate are made of SiO_2_, as illustrated in Figure 1. The essential origin of VD in our system is the asymmetric spatial coupling between this intensity distribution and the three-dimensional profile of the spiral.

Unlike conventional CD, which stems from spin chirality coupling, the VD effect here originates from the interplay between the wavefront’s spatial topology and the helical structure. A key manifestation is that the strong scattering occurs when the handedness of the incident vortex beam is opposite to that of the chiral structure. For instance, a left-handed spiral exhibits a gigantic scattering intensity under RHW illumination, and vice versa. Conversely, same-handed combinations yield markedly weaker responses. This handedness-dependent scattering is governed by a spatial coupling condition. When the high-intensity vortex beam is most coupled with the spiral metamaterials and least coupled with another symbol, a strong scattering asymmetry will occur. The scattering contrast between the two OAM states (+*l* and −*l*) constitutes the essence of the VD effect, which can be defined as [15],

(1) $$\mathrm{VD}=I_{+l}-I_{-l}$$

where $I_{\pm l}$ represent the scattering intensities under illumination by RHW and LHW, respectively. This mechanism enables chirality discrimination at a fixed wavelength without relying on polarization variation, highlighting the capability of OAM beams to probe and manipulate three-dimensional chiral light-matter interactions.

The chiral metamaterials demonstrated here are modeled as a continuous three-dimensional spiral winding around the *z*-axis. Its path is parameterized by

(2) $$\left\{\begin{array}{c} x(\theta )=R\cos\theta \\ y(\theta )=R\sin\theta \\ z(\theta )=p\theta /2\pi \end{array}\right.$$

where the rotation angle $\theta \in \left[0,2\pi N\right]$, the spiral radius *R* = 7 μm, the pitch *p* = 5 μm, and the number of turns *N* = 1. The 3D geometry is further characterized by the structural pitch *p* and the spiral angle *α*, which are geometrically constrained by the radius *R* through the relation,

(3) $$\tan\alpha =\frac{p}{2\pi R}$$

The handedness of the spiral metamaterials is determined by the sign of the rotation angle *θ*. A uniform top layer with thickness *H_t_* = 0.5 μm follows the same spiral contour.

To investigate the VD induced by the interaction between the vortex beam and the designed chiral metamaterial, a Laguerre–Gaussian (LG) beam with either LHW or RHW is employed as the illumination source. The beam is normally incident at the location of 0.5 μm below the substrate of the chiral metamaterial. Its polarization is aligned along the *x*-axis to exclude any chiral contribution from SAM. Within the paraxial approximation, the electric field distribution of the LG vortex beam is described as [20],

(4) $$E^{in}\left(r,\varphi \right)=C\frac{r^{\left|l\right|}}{\sqrt{\left|l\right|!}}\exp\left(-\frac{r^{2}}{w_{0}^{2}}\right)\exp\left(il\varphi \right)x$$

where *C* is a normalized constant independent of *l* and *r*, and $w_{0}$ = 1.1 µm. The operation wavelength of the light source is fixed at 800 nm. Since SiO_2_ is highly transparent at the operating wavelength (800 nm), the SiO_2_ substrate has a negligible impact on the transmission and reflection. The refractive indices for SiO_2_ and Au are 1.44 and 0.18 + 5.1*i* respectively at 800 nm, which are from the data published by Edward Palik [21]. The intensity distribution of the vortex beam is,

(5) $$I(r,\varphi )=C^{2}\frac{r^{\left|2l\right|}}{\left|l\right|!}\exp\left(-\frac{2r^{2}}{w_{0}^{2}}\right)x^{2}$$

In our analysis, the beam waist *w_0_* remains fixed for all topological charges *l*, while the overall beam diameter expands as *l* increases. This configuration allows the spatial coupling between the vortex beam and the nanostructure to vary with *l*, which is essential for revealing the scale-dependent chiral response. Therefore, the LHW and RHW vortex beams have the same radius *r*, which increases with increasing topological charge |*l*|. All spectra are calculated by using the finite difference time domain Solutions, which is based on Lumerical FDTD Solutions (Ansys Inc., Canonsburg, PA, USA) using perfectly matched layer boundary conditions. Perfectly matched layer boundary conditions are adopted in the  ± *x*, ± *y*, and  ± *z* directions.

## 3. Results and Discussion

**Figure 2:**
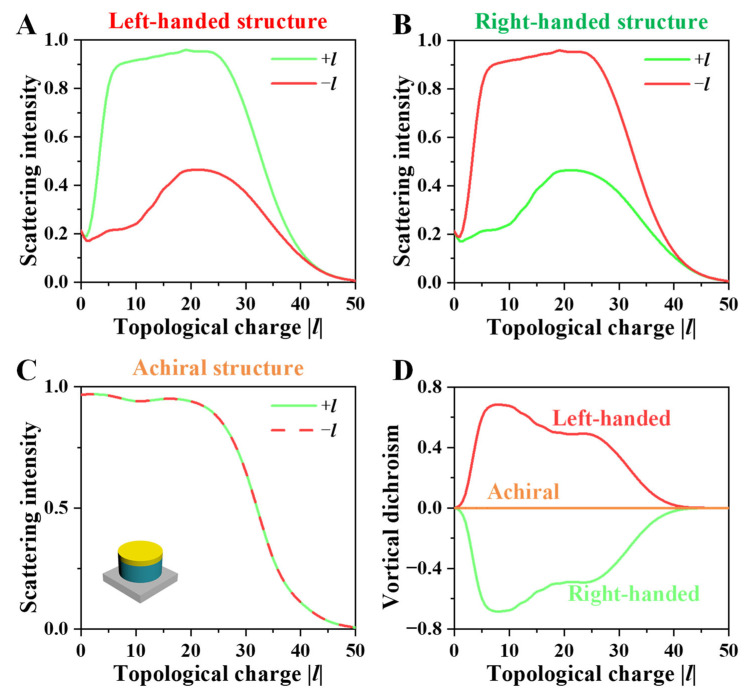
Scattering intensity spectra of (**A**) left-handed spiral, (**B**) right-handed spiral, and (**C**) achiral metamaterials illuminated by vortex beams with opposite topological charges (RHW, +*l* and LHW, −*l*). The left-handed and right-handed structures exhibit opposite scattering trends, while the achiral structure shows nearly identical responses for both beams. (**D**) Corresponding VD spectra of the three structures.

To verify the VD effect, we further analyze the scattering intensity spectra of the designed left-handed, right-handed, and achiral structures under illumination by vortex beams with opposite OAM states, as shown in Figure 2. The incident beams correspond to RHW (+*l*) and LHW (−*l*), and the scattering intensity represents the normalized backscattering intensity, which is total radiative output towards the backward direction. In Figure 2A, the left-handed structure exhibits a pronounced asymmetry between the two incident OAM states. The scattering intensity produced by RHW illumination is higher than that of LHW illumination across the broad topological charge range, indicating that the interaction between the left-handed structure and anti-helical beams is more effective. This strong OAM–matter interaction confirms that the scale of the helical path and handedness of the spiral metamaterials play a decisive role in the observed VD response. By contrast, Figure 2B shows that the right-handed structure displays the opposite trend. Under LHW illumination, the scattering intensity becomes dominant, while RHW excitation yields a substantially weaker response. This mirror-symmetric behavior between the two spiral metamaterials demonstrates that the sign of the OAM determines which handed structure produces stronger scattering. For reference, Figure 2C presents the response of an achiral structure incident by both LHW and RHW beams, which produces identical scattering intensities throughout the whole *l* range. The absence of any noticeable difference confirms that the VD effect arises solely from structural chirality rather than from the illumination conditions or material properties. As shown in Figure 2C, the scattering intensity of the achiral structure reaches nearly 100%. Due to the low topological charges (*l* < 25) of the vortex beams, the light field is concentrated near the center, leading to strong interaction with the structure. This high scattering response originates from a profound spatial mode mismatch between the incident OAM states and the structural geometry. Notably, as *l* increases beyond 25, the expanding central singularity of the vortex beam reduces its spatial overlap with the spiral metamaterials, thereby weakening the interaction and leading to a decline in scattering intensity. As illustrated in Figure 2D, the left- and right-handed structures exhibit opposite VD signs with gigantic magnitudes, reaching its maximum VD contrast of 0.69 at |*l*| = 9 within the investigated range, while the achiral structure maintains zero VD. These results clearly validate the existence of a strong OAM-dependent chiral response, confirming that the designed spiral metamaterials can achieve pronounced chiral VD effect.

**Figure 3:**
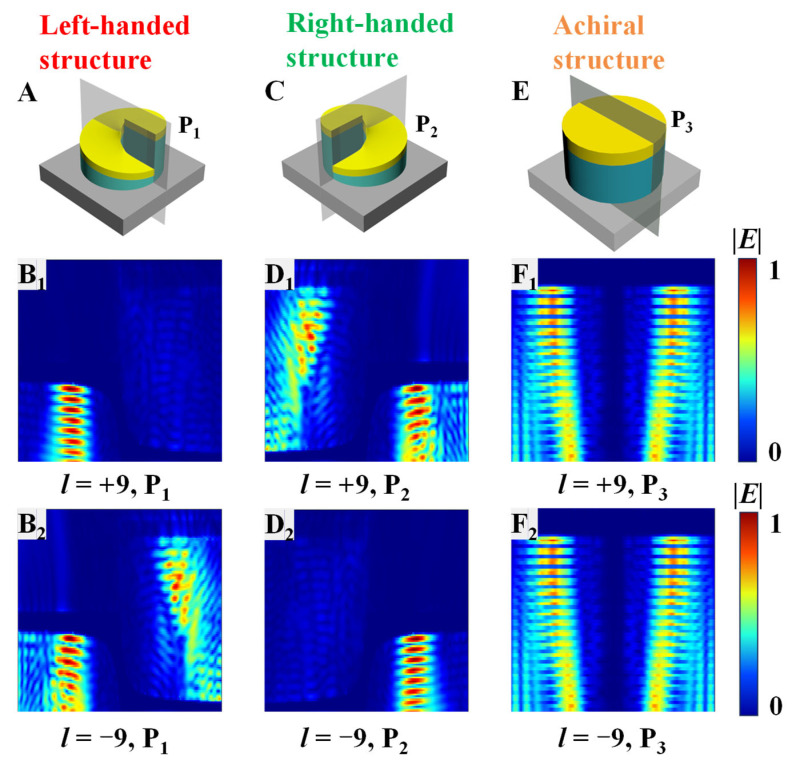
Schematics of (**A**) left-handed, (**C**) right-handed, and (**E**) achiral spiral metamaterials with cross-sectional planes P_1_, P_2_, P_3_, respectively. (**B_1_**,**D_1_**,**F_1_**) Electric-field intensity (|*E*|) distributions under RHW illumination (*l* = +9). (**B_2_**,**D_2_**,**F_2_**) Electric-field intensity (|*E*|) distributions under LHW illumination (*l* = −9).

To gain deeper insight into the physical origin of the VD observed in Figure 2, the electric-field distributions of the left-handed, right-handed, and achiral structures are analyzed under vortex-beam illumination. Examining the electric-field distribution allows us to directly visualize how the optical energy interacts with the spiral metamaterials. This visualization figure clarifies how such interaction leads to the large scattering asymmetry between opposite orbital angular momentum (OAM) states. Figure 3 displays the spatial field distributions for |*l*| = 9, where the VD response reaches its maximum. Panels (A–F) correspond to the cross-sectional planes P_1_, P_2_, and P_3_ for the three structures under RHW (+*l*) and LHW (−*l*) illumination.

In this work, we focus on the scattering response where the backscattering intensity is dominant. Under conditions of chiral match, the incident field excites the guided spiral modes to produce strong localized field enhancement, leading to decreased backscattering intensity. However, scattering in other directions makes its contribution to the VD effect negligible. For the left-handed structure [Figure 3(A,B_1_,B_2_)], the spatial mode mismatch prevents the vortex beam from efficiently coupling into the spiral dielectric channel under RHW (opposite-handed) illumination. Therefore, the incident energy is largely scattered by the metallic spiral. The scattered field generates a strongly enhanced and asymmetric electric field distribution on one side of the metamaterial. This enhanced near-field promotes efficient re-radiation, resulting in large backscattering intensity. In contrast, the vortex beam can penetrate and propagate along the spiral path inside the dielectric layer under LHW (same-handed) illumination. The energy is thereby distributed and guided throughout the spiral dielectric layer, which diverts energy away from the backscattering direction and results in substantially weaker scattering intensity. This difference in energy localization and propagation between RHW and LHW excitations directly accounts for the gigantic VD response observed at |*l*| = 9.

Furthermore, the field patterns exhibit similar spatial features but with reversed handedness for the right-handed structure [Figure 3(C,D_1_,D_2_)]. When illuminated by the LHW (opposite-handed) beam, the mode mismatch again leads to field localization and strong backscattering. Conversely, the RHW (same-handed) beam couples efficiently into the spiral channel, enabling guided propagation and lower scattering intensity. This reversed field behavior confirms that the energy coupling between the vortex beam and the spiral metamaterials is determined by their relative handedness, which is consistent with the mirror-symmetric scattering spectra shown in Figure 2.

For the achiral structure [Figure 3(E,F_1_,F_2_)], both RHW and LHW beams generate nearly identical electric-field distributions. The energy is symmetrically localized within the dielectric layer without any preferred circulation or directional confinement. This identical electric field response under opposite OAM states is a direct consequence of the structural symmetry, which dictates that the achiral structure cannot distinguish between RHW and LHW. It is noteworthy that the achiral structure also exhibits a uniformly distributed electric field within the dielectric layer, yet its scattering intensity remains high due to impedance mismatch. Therefore, a uniform field distribution does not necessarily imply reduced scattering. Instead, the decisive factor is the excitation of a chirality-dependent guided mode. For chiral spiral metamaterials, matching handedness between the vortex beam and the structure opens an efficient propagation pathway along the spiral dielectric channel, which diverts energy away from the scattering process. In contrast, the achiral structure lacks such a directional guiding mechanism. Despite the uniform field distribution, the energy is not preferentially guided out of the scattering region, resulting in stronger overall scattering. Consequently, the identical electric-field distributions for LHW and RHW lead to identical scattering spectra, which directly yields a near-zero VD response. This comparison demonstrates that the suppression of scattering in chiral systems results from the handedness-dependent energy guidance enabled by the spiral geometry. As a result, both OAM states produce uniformly strong scattering intensities across the spectrum, corresponding to a negligible VD response.

Overall, the field distribution at |*l*| = 9 clearly demonstrates that the VD effect originates from the handedness-dependent coupling efficiency into distinct electromagnetic modes, including intense interface reflection and guided spiral mode. When the handedness of the vortex beam is opposite to that of the spiral metamaterial, the spatial mode mismatch results in intense interface reflection and re-radiation that enhances backscattering. When the handedness of structure matches that of vortex beam, efficient coupling into a guided spiral mode suppresses scattering. This spatial mechanism provides a direct physical explanation for the asymmetric spectra observed in Figure 2.

**Figure 4:**
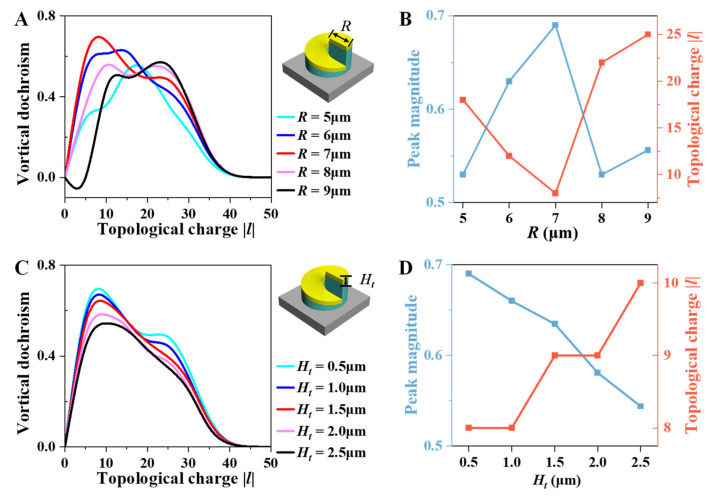
(**A**,**B**) Dependence of VD on the spiral radius *R*. (**C**,**D**) Dependence of VD on the metallic layer thickness *H_t_*.

To further investigate how the geometric parameters of the chiral structure influence the VD effect, we analyzed the dependence of VD effect on the spiral radius *R* and the gold spiral thickness *H_t_*, as shown in Figure 4. These results clarify the structural origin of the scale-dependent chiral response discussed above. Figure 4A,B illustrate the effect of the spiral radius *R* on VD. In Figure 4A, the VD spectra rise and then gradually decrease as the radius increases from *R* = 5 μm to *R* = 9 μm. The position of the VD peak shifts toward higher topological charge |*l*| as the *R* increases, indicating that larger spiral size couples more efficiently with higher-order vortex beams whose ring diameters are larger. The observed double-peak structure originates from the sequential spatial overlap between the expanding vortex ring and the different geometric boundaries of the three-dimensional spiral structure as the topological charge *l* increases. Figure 4B quantitatively shows the trend that both the VD peak magnitude (blue curve) and the corresponding |*l*| value (red curve) vary with the spiral radius. The maximum VD magnitude occurs at *R* = 7 μm, where the structural scale best matches the spatial profile of the vortex beam. This spatial coupling deteriorates for larger or smaller radii, leading to reduced coupling and weaker dichroism. Figure 4C,D show the effect of the gold-layer thickness *H_t_* on VD effect. In Figure 4C, the overall VD magnitude decreases gradually when *H_t_* increases from 0.5 to 2.5 μm. The peak value diminishes, and its position slightly shifts toward higher |*l*|. This suggests that thicker metallic spiral enhances absorption and weakens the chiral coupling between the optical vortex and the spiral channel. Furthermore, Figure 4D summarizes this dependence. As *H_t_* increases, the maximum VD magnitude (blue curve) declines monotonically, while the topological charge corresponding to the VD peak (red curve) shifts to higher |*l*|. In summary, both the spiral radius and gold-layer thickness significantly affect the VD response. These results confirm that VD effect is highly sensitive to the geometric scale of the chiral structure relative to the optical vortex, emphasizing the importance of size matching for optimizing chiral–OAM interactions.

Table 1 summarizes several representative chiral metamaterial designs that exhibit the VD effects reported in previous studies. Most of these structures, including Double-Elliptical metamaterials, multi-layer metamaterials, and so on, which show moderate VD magnitudes within limited range. In contrast, the spiral metamaterials proposed in this work achieve gigantic VD magnitude of 0.69. This result demonstrates the strong interaction between the optical vortex and the chiral metamaterials in our design, highlighting its superior chiroptical performance compared with previously developed metamaterials.

## 4. Conclusions

In conclusion, we have proposed three-dimensional spiral metamaterials that exhibit pronounced VD effect under vortex-beam illumination. The intrinsic chirality of the spiral metamaterials enables strong coupling between the OAM of light and the handedness of the structure, giving rise to markedly asymmetric scattering intensities for opposite topological charges. The proposed spiral metamaterials achieve gigantic VD magnitude of 0.69, demonstrating the robust OAM–chirality interaction mechanism. Electric-field analysis reveals that this asymmetry originates from the handedness-dependent coupling efficiency into distinct electromagnetic modes. Specifically, opposite handedness causes spatial mode mismatch and strong scattering, while matched handedness enables efficient coupling into a guided spiral mode, thereby diverting energy and suppressing scattering. This work not only provides direct physical insight into the spatial origin of VD effect but also offers an effective structural platform for designing high-performance chiral photonic devices and expanding the frontier of OAM-based light–matter interactions.

## Figures and Tables

**Table 1:** Performance comparison to other metamaterials.

Structure	VD Peak	Ref.
Double-Elliptical	0.21	[14]
Multi-layer metamaterials	0.38	[16]
Meta-molecules	0.48	[19]
Spiral metamaterials	0.69	This work

## Data Availability

The data presented in this study are available on request from the corresponding author.
